# Cross-Cultural Differences in Fear of Death, Emotional Intelligence, Coping with Death, and Burnout Among Nursing Students: A Comparative Study Between Spain and Portugal

**DOI:** 10.3390/bs15070993

**Published:** 2025-07-21

**Authors:** Miguel Ángel Martín-Parrilla, Noelia Durán-Gómez, Maria do Céu Marques, Casimiro Fermín López-Jurado, Margarida Goes, Macarena C. Cáceres

**Affiliations:** 1 Departamento de Enfermería, Centro Universitario de Plasencia, Universidad de Extremadura, 10600 Plasencia, Spain; miguelangelmp@unex.es; 2 Departamento de Enfermería, Facultad de Medicina y Ciencias de la Salud, Universidad de Extremadura, 06006 Badajoz, Spain; casimirolj@unex.es (C.F.L.-J.); mcaceres@unex.es (M.C.C.); 3 Escola Superior de Enfermagem São João de Deus, Universidade de Évora, 7000-869 Evora, Portugal; mcmarques@uevora.pt (M.d.C.M.); mgoes@uevora.pt (M.G.); 4 Comprehensive Health Research Center, Universidade de Évora, 7004-516 Evora, Portugal

**Keywords:** undergraduate students, nursing, nursing education, fear of death, emotional intelligence, coping skills, student burnout, mental health

## Abstract

Nursing students often face emotional challenges related to death and dying, affecting their psychological well-being, emotional intelligence, and academic success. Cultural and educational factors may influence how they experience and manage these stressors. This study aimed to compare fear of death, emotional intelligence, coping with death, and academic burnout among second-year nursing students in Spain and Portugal to identify cross-cultural differences. A cross-sectional study was conducted among 174 second-year nursing students from the University of Extremadura (Spain) and the University of Évora (Portugal) during the 2023/2024 academic year. The instruments used included the Collett–Lester Brief Fear of Death Scale, the Trait Meta-Mood Scale—24 ítems, the Bugen’s Coping with Death Scale, and the Maslach Burnout Inventory—Student Survey. Descriptive statistics were calculated, and group comparisons were performed using independent samples *t*-tests and Welch’s *t*-tests, depending on variance homogeneity. A two-way ANOVA was also conducted to explore interactions between gender and nationality. The Spanish sample comprised 86 women and 21 men, and the Portuguese sample included 58 women and 9 men. The mean age across the sample was 21.5 years (SD = 4.15). No significant differences were observed in fear of death or emotional intelligence between the two groups. However, Portuguese students showed significantly better coping with death, but also higher academic burnout and cynicism. Spanish students reported greater perceived efficacy. Although emotional intelligence and death anxiety were similar, Portuguese students demonstrated stronger coping skills but experienced more burnout. This suggests that coping alone may not buffer academic stress, emphasizing the need for culturally tailored interventions to enhance emotional readiness and reduce burnout.

## 1. Introduction

Nursing is a profession that demands not only technical expertise but also significant emotional resilience (Han et al., 2022). Nurses frequently encounter suffering, loss, and death, requiring them to develop coping strategies that allow them to provide compassionate care while maintaining their psychological well-being (Alodhialah et al., 2024). For nursing students, these challenges arise early in their training, often during their first clinical experiences, where they are exposed to distressing situations for the first time (Aryuwat et al., 2024). Their ability to manage emotions, cope with the reality of death, and withstand academic pressures plays a crucial role in shaping their future professional performance and personal well-being (Kunzler et al., 2020).

One of the most significant psychological factors affecting nursing students is the fear of death (FD), which can influence their attitudes toward end-of-life care, decision-making in critical situations, and emotional responses to patient mortality (Cybulska et al., 2022). Death, while a universal phenomenon, is perceived differently across cultures. Societal attitudes, religious beliefs, and personal experiences shape individuals’ fears and anxieties regarding mortality (Belak & Goh, 2024). In some cultures, death is openly discussed and ritualized, fostering a greater sense of acceptance, while in others, it remains a sensitive or even taboo topic (Hidalgo et al., 2020). These cultural perspectives likely influence nursing students’ fears and preparedness when dealing with dying patients and grieving families (Zahran et al., 2021).

Closely related to FD is the role of emotional intelligence (EI) in nursing practice (Aradilla-Herrero et al., 2013). EI, defined as the ability to recognize, understand, and regulate emotions in oneself and others (Salovey & Mayer, 1990), is essential for effective patient communication and stress management in clinical settings (Coronado-Maldonado & Benítez-Márquez, 2023). Nurses with high EI are better equipped to manage patient interactions, make sound clinical judgments under pressure, and maintain psychological well-being despite the emotional demands of their profession (Ayed, 2025). However, the extent to which nursing students develop EI may be influenced by cultural factors, such as norms regarding emotional expression and regulation (Dugué et al., 2021). In some cultures, emotional restraint is valued, leading students to internalize stress rather than express or process it, while in others, openness and emotional sharing are encouraged as coping mechanisms. These cultural variations may impact nursing students’ ability to regulate emotions effectively, affecting both their personal well-being and professional performance (Yang & Wang, 2019).

Beyond EI, nursing students must also develop effective coping mechanisms to manage death and dying. Unlike many other healthcare professionals, nurses are in direct contact with terminally ill patients and their families, making their ability to cope with loss a crucial skill (Nabirye et al., 2025). However, students often enter clinical training with little or no preparation for dealing with death, leading to significant emotional distress (Lisai-Goldstein & Shaulov, 2024). Coping strategies are influenced by a variety of factors, including previous exposure to death, personal beliefs, and cultural attitudes toward grief and loss (Martín-Parrilla et al., 2025). In some societies, structured grief rituals provide a framework for processing loss, while in others, grief may be more individualized and internalized (Aeschlimann et al., 2024).

Alongside these emotional and psychological challenges, nursing students also face significant academic pressures that can contribute to burnout. Academic burnout, characterized by emotional exhaustion, depersonalization, and a reduced sense of personal accomplishment, is a growing concern in nursing education (Hwang & Kim, 2022). The demanding nature of nursing programs, combined with clinical responsibilities and emotional strain, places students at high risk for burnout (Abram & Jacobowitz, 2020). While burnout is a global phenomenon, its prevalence and severity vary across cultural contexts due to differences in academic expectations, institutional support, and societal attitudes toward stress and resilience (Tating et al., 2023). In some cultures, high academic pressure is normalized, leading students to endure stress without seeking support, whereas, in others, mental health awareness and structured stress management strategies are more integrated into educational systems (Ross et al., 2023).

Spain and Portugal, despite their geographical proximity and shared historical connections, have different healthcare systems, educational structures, and cultural perspectives on death, emotion, and stress management. These differences reflect broader sociocultural patterns seen across Europe, where national traditions and values significantly shape end-of-life care practices and attitudes (Gysels et al., 2020), highlighting the importance of culturally sensitive approaches in healthcare. These differences may influence how nursing students from each country experience FD, EI, coping strategies, and academic burnout. Although both institutions involved in this study—the University of Évora (Portugal) and the University of Extremadura (Spain)—share a common goal in preparing competent nursing professionals, there are curricular differences that help contextualize the choice of second-year students. In Évora, students begin hospital placements in the fourth semester, while in Extremadura, these placements start in the fifth semester. This timing places second-year students in a transitional academic phase in both institutions, prior to clinical exposure. By examining these psychological and emotional factors among nursing students in Spain and Portugal, this study aims to contribute to a deeper understanding of how cultural and educational contexts shape the experiences of future nurses. Insights from this research can inform nursing curricula, helping educators develop targeted interventions to enhance students’ emotional resilience, preparedness for end-of-life care, and overall well-being.

### Purpose

This study aims to compare FD, EI, coping with death (CD), and academic burnout among second-year nursing students from Spain and Portugal, identifying potential cross-cultural differences and their implications for nursing education and professional preparedness.

## 2. Materials and Methods

### 2.1. Design

This study employed a cross-sectional design to explore psychological and emotional variables among nursing students prior to clinical exposure. The observational nature allowed for the examination of naturally occurring differences without experimental manipulation.

### 2.2. Study Population and Setting

The research sample comprised undergraduate Nursing students from the University of Extremadura (Spain) and the University of Évora (Portugal) during the 2023/2024 academic year, selected through convenience sampling. Eligibility criteria required students to be enrolled in the second year of the Nursing program and to provide informed consent. Excluding students with prior clinical experience allowed us to focus on participants before their first hospital placements, providing insight into their baseline emotional and psychological state. This approach aimed to identify formative needs and vulnerabilities prior to clinical exposure, ensuring a more homogeneous sample and reducing the impact of external factors on the results.

Data collection in both institutions was conducted prior to the beginning of students’ clinical placements. Specifically, data collection took place at the University of Évora at the end of the third semester (December 2023) and at the University of Extremadura at the beginning of the fourth semester (February 2024).

The questionnaire was self-administered and distributed online via institutional platforms, ensuring accessibility and confidentiality. Participation was entirely voluntary and promoted through academic coordinators and classroom announcements. No incentives or compensation were offered.

All participants successfully completed the required forms and met the inclusion criteria, with no exclusions necessary.

### 2.3. Instruments and Measures

A sociodemographic questionnaire was specifically designed for this study to collect information on participants’ age, gender, and academic background, including grades and clinical experience.

The Collett–Lester Brief Fear of Death Scale (BFDS) was employed to assess students’ fears related to death and the dying process, both in relation to themselves and others. The BFDS is structured into four subscales, each containing seven items, totalling 28 items. Participants rated their responses using a Likert-type scale ranging from 1 (not at all) to 5 (a great deal). Both total scores and subscale scores were computed to determine the extent of fear associated with death and dying. The Spanish and Portuguese versions of this instrument have demonstrated strong psychometric properties, ensuring high reliability, validity, and internal consistency (Neto et al., 2025; Tomás-Sábado et al., 2007).

The Trait Meta-Mood Scale (TMMS-24), adapted from the Trait Meta-Mood Scale developed by Salovey and Mayer, was used to assess participants’ EI, particularly their ability to identify and regulate emotions. This scale focuses on three core dimensions: “emotional attention,” “emotional clarity,” and “emotional repair.” Standardized scores indicate gender-based differences in these dimensions. For emotional attention, optimal scores range from 22 to 32 for men and 25 to 35 for women. In terms of emotional clarity, the reference values are 26 to 35 for men and 24 to 34 for women. Regarding emotional repair, adequate scores range from 24 to 35 for men and 24 to 34 for women. The Spanish and Portuguese adaptation of the TMMS-24 has been validated with solid psychometric characteristics, confirming its reliability, validity, and internal consistency (Gonzalez et al., 2020; Queirós et al., 2005).

The Bugen’s Coping with Death Scale (CDS) was utilized to evaluate students’ ability to cope with death and their preparedness for facing it. This instrument consists of 30 items rated on a Likert-type scale from 1 (strongly disagree) to 7 (strongly agree). The total competence score is calculated by summing all item responses, with certain items (1, 13, and 24) being reverse-scored. The overall score ranges from 30 to 210. The Spanish and Portuguese adaptation of this scale has shown excellent psychometric properties, ensuring high levels of reliability, validity, and internal consistency (Camarneiro & Gomes, 2015; Galiana & Oliver, 2017).

The Maslach Burnout Inventory—Student Survey (MBI-SS) was applied to measure academic burnout among students. It assesses their levels of physical and mental exhaustion, cynicism, and self-efficacy regarding academic performance. The MBI-SS comprises three subscales, each with a different number of items. Participants respond using a 7-point frequency scale ranging from 0 (never) to 6 (always) to reflect their experiences. The Spanish and Portuguese versions of the MBI-SS have been tested and confirmed to possess strong psychometric properties, demonstrating high reliability, validity, and internal consistency (Duarte et al., 2012; Pérez-Fuentes et al., 2020).

### 2.4. Statistical Analysis

For data processing, we utilized version 2.3 of the Jamovi statistical software (The Jamovi Project, 2022). Initially, Levene’s test was conducted to assess the homogeneity of variances, and the Shapiro–Wilk test was applied to evaluate the normality of the distribution of continuous variables. Based on the results of these assumption checks, appropriate inferential tests were selected: when both normality and homogeneity of variances were met, the independent sample Student’s *t*-test was used; when normality was met but homogeneity of variances was violated, Welch’s *t*-test was applied; and when the assumption of normality was not satisfied, the non-parametric Mann–Whitney U test was employed. Additionally, a chi-square test (χ^2^) was used to compare gender proportions between groups, and a two-way ANOVA was conducted to assess age differences across gender and nationality. For all analyses, the α-level was set at *p* < 0.05.

## 3. Results

The study sample consisted of 174 second-year nursing students from Spain and Portugal, with 144 women (82.8%) and 30 men (17.2%). The overall mean age of participants was 21.54 years (SD = 4.15). The Spanish cohort included 107 students (21 men and 86 women), and the Portuguese cohort comprised 67 students (9 men and 58 women). Descriptive statistics for age across groups and genders are presented in Table 1.

A two-way ANOVA confirmed that the groups were also homogeneous in terms of age across gender and country (*p* = 0.178).

The following descriptive statistics shown in Table 2 provide an overview of the psychological and emotional variables across both groups, highlighting potential differences in FD, EI, CD, and burnout between nursing students from Spain and Portugal. Independent sample *t*-tests were performed to assess the significance of these differences.

Table 2 summarizes the mean scores and standard deviations of FD, EI, coping strategies, and academic burnout dimensions for Spanish and Portuguese nursing students. No significant differences were observed in FD and EI between the two groups. Portuguese students scored significantly higher in CD than Spanish students (*p* < 0.05). Regarding academic burnout, Portuguese students reported higher mean scores for exhaustion and cynicism and lower scores for academic efficacy compared to Spanish students, with these differences reaching statistical significance (*p* < 0.001).

**Figure 1:**
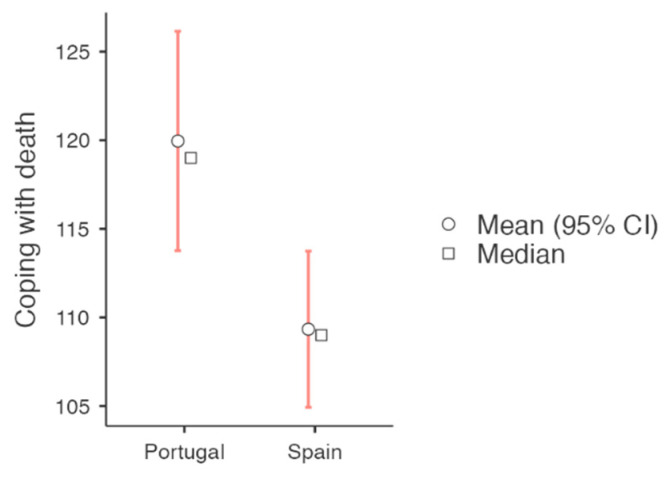
Descriptive plots of the Bugen’s CDS of both groups.

**Figure 2:**
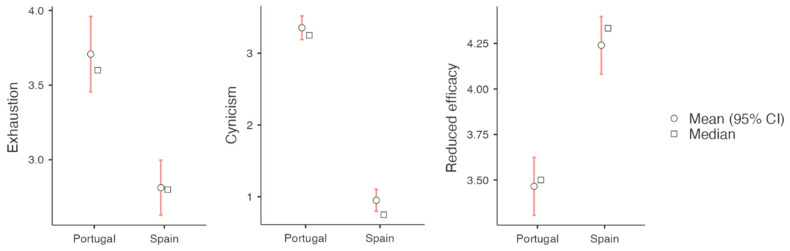
Descriptive plots of the MBI-SS dimensions of both groups.

For those pairs where differences were found between the Spanish and Portuguese groups, Figure 1 and Figure 2 present the visual representation of these differences.

Figure 1 visually displays the higher coping strategy scores observed in the Portuguese group. Figure 2 illustrates the burnout dimensions, showing that Spanish students reported comparatively better levels of burnout, with lower exhaustion and cynicism scores and higher academic efficacy.

## 4. Discussion

This study examined cross-cultural differences in FD, EI, CD, and academic burnout among second-year nursing students from Spain and Portugal. The findings revealed significant differences in CD and academic burnout, highlighting potential sociocultural and educational influences on nursing students’ emotional preparedness and stress management.

It is important to note that both the Spanish and Portuguese samples had a low proportion of male participants. However, as highlighted in previous research, this is a common challenge in nursing studies, as the profession continues to be predominantly female (Prosen, 2022), making it difficult to achieve balanced gender representation in study samples.

Regarding FD, no significant differences were observed between Spanish and Portuguese students, suggesting a shared perception of mortality. This could be attributed to cultural similarities between the two countries, including common traditions surrounding death and bereavement (Neto et al., 2025; Tomás-Sábado et al., 2007). While there are cultural differences between the two countries in broader sociocultural terms, these distinctions may not strongly influence fear of death among nursing students prior to clinical exposure. Prior research in Iberian populations has indicated that cultural and religious factors shape attitudes toward death, potentially leading to a comparable emotional response in nursing students (Cordero et al., 2018; Maestro-González et al., 2025; Pérez-De la Cruz, 2021). Furthermore, the results indicate that nursing students generally exhibit moderate levels of FD, a trend consistent with previous studies in healthcare education. This suggests that while death remains an emotionally charged concept, it does not provoke extreme anxiety in this population. Prior studies have pointed out that healthcare students, particularly those in nursing, often develop a balanced emotional response to death, shaped by their professional identity and exposure to discussions surrounding mortality (Almeida-Santos et al., 2022; Monsivais & Nunez, 2022).

In terms of EI, although no statistically significant differences emerged between the two groups, Portuguese students demonstrated slightly higher scores in emotional regulation. This subtle variation may reflect differences in educational training (Visiers-Jiménez et al., 2022), cultural approaches to emotional processing, or personal coping mechanisms (Laranjeira et al., 2021). Overall, the results indicate that nursing students in both countries exhibit adequate levels of EI, which is consistent with previous studies on healthcare students (Aljarboa et al., 2022; Galanis et al., 2024; Jabeen et al., 2024). EI plays a crucial role in psychological resilience, stress management, and effective patient interactions. Moderate to high levels of EI suggest that these students possess the capacity to regulate emotions and navigate emotionally demanding situations without being excessively overwhelmed (Aljarboa et al., 2022; Galanis et al., 2024; Jabeen et al., 2024).

A significant cross-cultural difference was found in CD, with Portuguese students reporting a higher capacity for coping compared to their Spanish counterparts. This disparity may stem from variations in nursing curricula (Antão et al., 2025; Visiers-Jiménez et al., 2021), including differences in exposure to palliative care training, death education, or clinical practice opportunities. In the University of Évora, students are introduced to subjects related to mental health, human development across the lifespan, and health–illness processes from the first to fourth semesters of the program, whereas at the University of Extremadura, similar content is generally covered starting in the fourth semester. These differences in curricular timing may contribute to the greater reported ability to cope with death among Portuguese students. Previous studies suggest that structured educational programs focused on end-of-life care can enhance students’ coping skills and preparedness for working with terminally ill patients (Yoong et al., 2023).

The most pronounced differences emerged in academic burnout, with Portuguese students reporting significantly higher levels of exhaustion and cynicism, along with lower academic efficacy. In contrast, Spanish students exhibited moderate-to-high exhaustion, lower cynicism, and moderately low academic efficacy. These findings suggest distinct psychological responses to academic stress between the two groups, which may be influenced by differences in coping strategies, resilience, or institutional factors such as workload distribution and support systems (Gómez-Urquiza et al., 2023). Another possible explanation lies in the academic methodologies employed at each institution. The University of Extremadura incorporates active learning strategies such as gamification (e.g., escape rooms), flipped classrooms, clinical simulation, and problem- and project-based learning, all of which are explicitly outlined in course syllabi. These student-centered approaches may enhance engagement and motivation, thereby mitigating academic burnout. In contrast, although the University of Évora has a well-established clinical simulation unit, there is limited evidence of the systematic use of innovative pedagogical methods in theoretical classes, which may reduce opportunities to buffer emotional exhaustion.

The elevated exhaustion and cynicism observed among Portuguese students suggest a more pronounced emotional detachment and a heightened perception of academic stress, which aligns with prior research linking these dimensions of burnout to psychological distress and decreased motivation (Salgado & Au-Yong-oliveira, 2021). Moreover, their lower sense of academic efficacy could indicate feelings of incompetence or diminished confidence in their professional preparedness, which may exacerbate emotional exhaustion and contribute to a cycle of disengagement (Rodeles et al., 2025).

On the other hand, while Spanish students also experienced notable levels of exhaustion, their lower cynicism and slightly higher academic efficacy suggest a comparatively more adaptive psychological response. This pattern may reflect a greater sense of purpose or engagement, potentially buffering against the most detrimental effects of burnout (Vizoso et al., 2019). However, the combination of moderate-to-high exhaustion and low academic efficacy still raises concerns, as both factors are predictors of long-term emotional strain and professional dissatisfaction in healthcare fields (Garcia et al., 2019; Nagle et al., 2024).

### 4.1. Implications for Nursing Education and Practice

These findings underscore the necessity of integrating comprehensive emotional and psychological training into nursing education. Given the observed cross-cultural differences, particularly in CD and burnout, educational institutions should consider curriculum enhancements that incorporate structured death education, EI training, and resilience-building strategies. Moreover, addressing academic burnout requires a multifaceted institutional approach, including optimizing course structures, providing mental health resources, and fostering a supportive academic environment.

From a clinical perspective, fostering EI and coping skills in nursing students can have profound implications for patient-centered care, communication in end-of-life scenarios, and overall professional well-being, as previous studies have already suggested (Ayed, 2025; Martín-Parrilla et al., 2025; Raghubir, 2018; Wong, 2025).

Future research should focus on longitudinal assessments of how these psychological factors evolve throughout nursing education and how targeted interventions can enhance students’ preparedness for the emotional challenges of clinical practice.

### 4.2. Limitations

While the cross-sectional design allows for the identification of associations between psychological and emotional variables, it does not permit causal inferences. Future research employing longitudinal designs could provide deeper insights into how nursing students’ attitudes toward death and coping strategies evolve throughout their education and clinical experiences.

While the questionnaires were administered to the majority of second-year students at both institutions—thereby minimizing selection bias—the generalizability of the findings remains limited. This study specifically targeted nursing students prior to their clinical exposure, whose perceptions may differ significantly from those of practicing nurses or students at different stages of their training. Additionally, the absence of socioeconomic data limits our ability to control for or interpret the influence of contextual factors such as income level, employment status, or family responsibilities, which may also affect emotional responses and coping mechanisms. Consequently, caution should be exercised when extrapolating these results beyond similar academic settings.

Despite these limitations, this study contributes valuable evidence to the field by providing a comparative perspective between Spanish and Portuguese nursing students, which is rarely explored in the literature. The use of well-established psychological assessment tools ensures methodological rigor, and the findings offer relevant implications for nursing education.

## 5. Conclusions

This study examined FD, EI, CD, and academic burnout among second-year nursing students from Spain and Portugal, identifying both similarities and differences. No significant differences were found in FD or EI, suggesting that students in both countries share similar emotional profiles. These results align with existing literature, indicating that moderate levels of FD and stable EI are common among nursing students at this stage of training.

However, Portuguese students reported higher competence in CD but also greater exhaustion and cynicism than their Spanish counterparts. This suggests that feeling more prepared for death-related situations does not necessarily protect against burnout. These findings highlight the need for nursing curricula to balance emotional resilience training with strategies to prevent burnout.

## Figures and Tables

**Table 1:** Descriptive statistics of the sociodemographic questionnaire.

Group	Gender	N (%)	*p*-Value	Mean Age (SD)	*p*-Value
ESP	Male	21 (19.63)	0.293	20.86 (2.35)	0.255
	Female	86 (80.37)		21.88 (4.22)	
	Total	107 (100)		21.68 (3.93)	
PRT	Male	9 (13.43)		21.67 (4.89)	
	Female	58 (86.57)		21.08 (4.12)	
	Total	67 (100)		21.13 (4.22)	

**Table 2:** Descriptive statistics and independent sample *t*-tests of all psychological and emotional variables across both groups.

	Mean_ESP_ (SD)	Mean_PRT_ (SD)	Mean Difference	*t*-Test	*p*-Value
Collett–Lester’s BFDS					
Fear of One’s Own Death	2.95 (0.96)	2.88 (1.09)	−0.069	Mann-Whitney U ^a^	0.647
Fear of the One’s Dying	3.21 (0.90)	3.07 (1.10)	−0.145	Mann-Whitney U ^a^	0.408
Fear of Death of Others	3.64 (0.76)	3.41 (0.88)	−0.226	Mann-Whitney U ^a^	0.088
Fear of the Dying of Others	3.35 (0.71)	3.35 (0.97)	0.003	Welch’s t ^b^	0.985
TMMS-24					
Emotional attention	28.81 (4.95)	28.90 (6.79)	0.082	Mann-Whitney U ^a^	0.587
Emotional clarity	28.03 (6.64)	26.57 (6.61)	−1.461	Student’s t	0.159
Emotional repair	27.50 (5.65)	28.00 (6.30)	0.505	Student’s t	0.584
Bugen’s CDS					
Coping with death	109.34 (23.25)	119.96 (25.85)	10.619	Welch’s t ^b^	0.007
MBI-SS					
Exhaustion	2.81 (0.97)	3.71 (1.06)	0.894	Student’s t	<0.001
Cynicism	0.95 (0.81)	3.35 (0.69)	2.404	Mann-Whitney U ^a^	<0.001
Reduced efficacy	4.24 (0.84)	3.47 (0.66)	−0.775	Mann-Whitney U ^a^	<0.001

H_1_: μ_PRT_ ≠ μ_ESP_. ^a^ A low *p*-value (*p* < 0.05) in the Normality Test (Shapiro–Wilk) suggests a violation of the assumption of normality. ^b^ Levene’s test is significant (*p* < 0.05), suggesting a violation of the assumption of equal variances.

## Data Availability

The data presented in this study are available upon request from the corresponding author due to ethical reasons.

## Abbreviations

The following abbreviations are used in this manuscript:

BFDS	Brief Fear of Death Scale
CD	Coping with Death
CDS	Coping with Death Scale
EI	Emotional Intelligence
ESP	Spain
FD	Fear of Death
MBI-SS	Maslach Burnout Inventory—Student Survey
PRT	Portugal
SD	Standard Deviation
TMMS-24	Trait Meta-Mood Scale—24 ítems
